# Rapidly progressive glomerulonephritis in two Zambian children: a case report

**DOI:** 10.11604/pamj.2022.42.21.33318

**Published:** 2022-05-10

**Authors:** Chisambo Mwaba, Chibamba Ngomalala Mumba, Musyani Simukonde, Somwe Wa Somwe

**Affiliations:** 1 Department of Paediatrics and Child Health, School of Medicine, University of Zambia, Lusaka, Zambia,; 2 Department of Pathology and Microbiology, School of Medicine, University of Zambia, Lusaka, Zambia

**Keywords:** Acute kidney injury, rapidly progressive glomerulonephritis, crescentic glomerulonephritis, renal disease, case report

## Abstract

Rapidly progressive glomerulonephritis (RPGN) is a rare syndrome which is marked by a sudden rise in serum creatinine and the presence of crescents on renal biopsy. If appropriate and timely treatment is not instituted, as many as 90% of affected patients may develop End Stage Renal Disease (ESRD). There is only limited access to renal replacement therapy in many low resource countries, thus it is important that awareness of this entity is raised. We narrate the clinical course of two children who were admitted with rising serum creatinine, hypertension and haematuria and who were subsequently diagnosed with crescentic glomerulonephritis on biopsy. Despite having received immunosuppressive therapy, both children had a poor renal outcome, perhaps due to delays in institution of appropriate treatment. It is imperative that all clinicians who manage children are made aware of this clinical syndrome so that timely referrals to nephrology are done. This will help to improve renal outcomes.

## Introduction

Rapidly progressive glomerulonephritis (RPGN) is a nephrology emergency that presents with a sudden and progressive rise in serum creatinine that occurs within days to weeks and results into end stage renal disease (ESRD) in up to 50-90% of patients if not treated in a timely manner [1-3]. It is associated with the histological pattern known as crescentic glomerulonephritis (CGN) [1]. A patient is said to have CGN when the biopsy specimen exhibits more than 50% of glomerular crescents. This cut-off definition is not universally adopted [1]. Rapidly progressive glomerulonephritis (RPGN) and CGN are usually considered to be synonymous but other entities such as haemolytic uremic syndrome (HUS), tubulo-interstitial nephritis and proliferative glomerulonephritis can also present with a similar clinical picture. A histological diagnosis is therefore imperative [3]. Patients with CGN usually present with the nephritic syndrome (haematuria, oedema, hypertension, renal dysfunction) but on occasion children may also present with nephrotic syndrome (oedema, hypo-albuminaemia, and proteinuria). Even though the typical patient has a rapidly rising serum creatinine, there have been a few reports describing a more indolent clinical course [3,4]. Treatment can improve outcomes if instituted early. This usually includes the administration of 3-5 daily doses of high dose methylprednisolone followed by either Cyclophosphamide or Mycophenolate Mofetil. Plasmapheresis may also be administered depending on the underlying condition [1]. Even though CGN has a low incidence, it is imperative that it is recognised early because of the higher risk of ESRD in untreated patients [5]. This is especially so in low resource settings where access to renal replacement therapy (RRT) is limited. We report on the clinical course of two children who presented with the RPGN syndrome and who were diagnosed with CGN on biopsy at the University Teaching Hospital Children´s Hospital (UTH-CH) paediatric nephrology unit in Lusaka, Zambia.

## Patient and observation

**Patient 1**

**Patient information:** an eleven-year-old male who had previously been well, presented with a three-week history of painless macroscopic haematuria, reduced urine output and body swelling. He denied any history of skin rash, painful, swollen joints or haemoptysis. There was no family history of renal disease.

**Clinical findings:** on physical examination he had anasarca and blood pressure (BP) of 150/100 mmHg (systolic > 95^th^ centile, diastolic 99^th^ centile). Bedside urine dipstick showed blood 3+ and protein 2+.

**Timeline of current episode:** June 2019: child had macroscopic haematuria and swelling of face and body for three weeks before seeking medical care. July 2019: patient admitted at UTH-CH and intravenous methylprednisolone given July 2019: renal biopsy performed and MMF commenced August 2019: discharged home and advised to return for follow-up.

**Diagnostic assessment:** the initial laboratory findings were as follows: serum creatinine- 219 μmoles/L, urea-30 mmol/L, serum sodium-139 mmol/l, serum potassium-3.37 mmol/l, haemoglobin-9.6 g/dl, platelets-701 x 10^9^/L, serum protein-65g/dl, albumin-not done. There were no blasts or schistocytes on the peripheral smear, and the solubility test was negative. Additionally, the infectious screen was negative for both hepatitis B and syphilis. The autoimmune screen indicated normal levels of complement protein 3 and 4, negative Anti-Nuclear Antibodies (ANA) and negative anti-neutrophil cytoplasmic antibodies (ANCA). Anti-streptolysin Titres (ASOT) and anti-DNase B were not tested due to limited resources. Imaging studies were conducted. The kidney ureter bladder ultrasound (KUB U/S) showed normal-sized kidneys with increased echogenicity and preserved cortico-medullary differentiation. The chest radiograph (CXR) was normal, while echocardiography demonstrated a normal cardiac structure and an ejection fraction (EF) of 65%. Initially a diagnosis of acute post-infectious glomerulonephritis possibly post-group A streptococcal glomerulonephritis was made, and conservative management was instituted. However, there was a noted worsening in the patient´s clinical condition; the oedema increased, and she developed bilateral pleural effusions. Blood tests revealed a decrease of the serum albumin to 16 g/dl and a rise of the serum creatinine to 302 μmoles/. In addition, the platelet count dropped to only 70 x 10^9^/L, while the haemoglobin decreased to 2.5 g/dl. The total serum bilirubin remained normal at 12.5 μmol/L while repeat peripheral smear showed 1+ of schistocytes. We had no facility to test for haptoglobulins.

**Figure 1:**
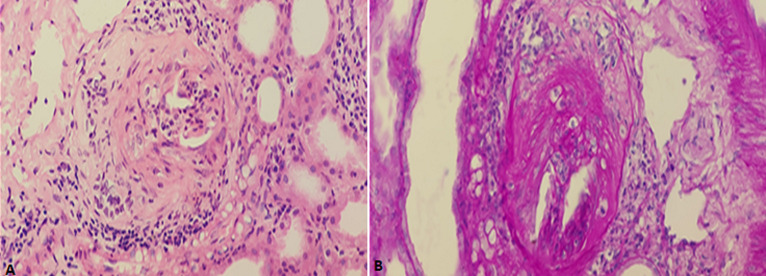
needle biopsy of the kidney demonstrating: A) (x400 hematoxylin and eosin stain); B) (x400 periodic acid Schiff stain); A) fibro-cellular crescent; B) glomerulus with a capillary tuft that is collapsed crescent; B) glomerulus with a capillary tuft that is collapsed

**Diagnosis:** rapidly progressive glomerulonephritis secondary to CGN or atypical HUS were considered as possible diagnoses. Once the platelet count normalised an ultrasound guided percutaneous kidney biopsy was performed. The renal biopsy showed a total of 14 glomeruli with more than 50% demonstrating crescents, mostly cellular and a few fibrocellular (Figure 1). The glomeruli demonstrated hypercellularity with a neutrophil infiltrate, while the interstitium showed mild tubular atrophy and simplification of the tubules. Given the histology and clinical presentation, a histological diagnosis of crescentic glomerulonephritis arsing on a background of post infectious glomerulonephritis was made.

**Therapeutic interventions:** five doses of intravenous methylprednisolone at 10 mg/kg/day were given, followed by oral prednisolone at 2 mg/kg for a month before weaning the dose. Mycophenolate, Mofetil at an initial dose of 300 mg/m^2^/dose twice daily, was also commenced after biopsy confirmed CGN. Intravenous cyclophosphamide was not available.

**Follow-up and outcome of interventions:** the patient recovered with improvement in renal function (serum creatinine-112 μmol/L) and resolution of the oedema and ascites over a one-month period. He was discharged home and scheduled for a two-week review at University Teaching Hospital Children´s Hospital (UTH-CH).

**Patient perspective:** “my son was very unwell, and I thought that he would never recover. Now the swelling has disappeared and he is able to play again. I hope that with time the condition will be completely cured”

**Informed consent:** written informed consent was obtained from the legal guardian of the child for publication of the case report and attached images. A copy of the written consent is available.

**Patient 2**

**Patient information:** a six-year-old girl presented to a district hospital with a 2-week history of body swelling. She did not have gross haematuria, arthritis or skin rash. There was nothing significant in her past medical history. A diagnosis of nephrotic syndrome was made and treatment with 2 mg/kg of prednisolone and enalapril was commenced. Two weeks later it was noted that the child´s serum creatinine was 243 μmol/L while the serum urea was 20.5 mmol/L. She was referred to our facility for management of the new onset acute kidney injury (AKI).

**Clinical findings:** on admission, she had anasarca, pulmonary oedema and BP169/109 mmHg (systolic > 99^th^ centile, diastolic > 99 centile), urine dipstick blood 3+ and protein 2+.

**Timeline of current episode:** March 2019: swelling of face and body for two weeks. March 2019: patient admitted at a district hospital for two weeks. April 2019: admitted at UTH-CH, received intravenous methylprednisolone at 10 mg/kg/day for five days and peritoneal dialysis was administered for three weeks. May 2019: renal biopsy performed and parents counselled about poor prognosis. Child discharged to provincial hospital for palliation.

**Diagnostic assessment:** her venous blood gas result showed: potassium-9 mmol/L, sodium-139 mmol/L, ionised calcium-0.75 mmol/L. The blood results showed: haemoglobin-5.6g/dl, platelets-215 x 10^9^/L, urea-47 mmol/L, creatinine-1079 μmol/L, serum albumin 33.8 g/dl. Due to financial constraints, we were unable to perform an autoimmune screen. Her chest radiograph showed pulmonary oedema while the kidney ureter bladder ultrasound (KUB) showed increased renal echogenicity.

**Diagnosis:** in view of the rapidly rising creatinine, a diagnosis of AKI secondary to RPGN with severe fluid overload and hyperkalaemia was made. The renal biopsy was performed 3 weeks later. Histologically, 10 glomeruli were demonstrated. Of these glomeruli, 6 had fibro cellular crescents, 1 globally sclerosed and 3 with mesangial expansion. The interstitium had a moderate chronic inflammatory infiltrate. The tubules demonstrated mild to moderate atrophy and signs of previous injury. The blood vessels were within normal limits. Given the clinical picture and histologic findings, a diagnosis of crescentic glomerulonephritis was made. The primary lesion or cause was not established.

**Therapeutic interventions:** peritoneal dialysis (rapid flushes with 4 L of 1.5% fluid followed by 24 cycles with 1.5% peritoneal dialysis fluid (higher strength fluids were out of stock) was instituted and intravenous methylprednisolone at 10 mg/kg/day (five doses) was given. This was followed by prednisolone 2 mg/kg/day orally. In addition, a calcium channel blocker (Nifedipine) and diuretics were administered. Once dialysis had been done for 24 hours, packed red blood cells at 10ml/kg were also transfused. Renal biopsy was deferred until the blood pressure had been controlled and the patient´s general condition had improved.

**Follow-up and outcome of interventions:** the prognosis for dialysis free survival for this patient was poor and since the family did not meet socio-economic criteria for RRT, the patient was discharged home for palliative care. She died three weeks later.

**Patient perspective:** “it´s hard to believe that my daughter died so unexpectedly. She was always a healthy child. It´s unfortunate that medicines and treatments that could have kept her alive are not available, where we live.”

**Informed consent:** written informed consent was obtained from the legal guardian of the child for publication of the case report. A copy of the written consent is available.

## Discussion

Typically CGN presents as a nephritic syndrome with rapidly decreasing glomerular filtration rate (GFR). On occasion patients may develop a nephrotic range proteinuria with overt nephrotic syndrome as for case 1 and this is associated with a poorer prognosis. Other clinical features associated with worse prognosis are a higher serum creatinine at presentation as was the case for patient 2, and prolonged duration of illness prior to nephrology contact as was the case once again for patient 2 [1]. Without treatment poor renal outcome has been reported in as many as 80-90% of patients [5]. With treatment, the proportion of children with ESRD range from 27% to 50% [5]. The predictors of a poor renal outcome include older patient age, underlying disease e.g. ANCA associated vasculitis, and in some series post-infectious aetiology [6]. Outcome is particularly improved by clinicians having a high index of suspicion and making an early diagnosis and referral to nephrology for an accurate clinical-pathologic diagnosis, appropriate treatment and follow up [1,3].

## Conclusion

Renal replacement therapy is not widely available in Africa largely due to limited economic resources and scarcity of trained manpower, thus a diagnosis of ESRD is a virtual death sentence [7]. These two cases illustrate that since ESRD secondary to RPGN can be prevented by timely and appropriate treatment, it is imperative that awareness among clinicians is raised to facilitate a heightened index of suspicion which will result in earlier referrals to nephrology.
